# (Acetato-κ*O*)diaqua­[2-(1*H*-benzotriazol-1-yl)acetato-κ*O*](1,10-phenanthroline-κ^2^ *N*,*N*′)manganese(II) dihydrate

**DOI:** 10.1107/S1600536812007404

**Published:** 2012-02-24

**Authors:** Ling Zeng

**Affiliations:** a College of Chemistry and Chemical Engineering of Bohai University, Jinzhou, Liaoning 121000, People’s Republic of China

## Abstract

In the hydrated title complex, [Mn(C_8_H_6_N_3_O_2_)(CH_3_CO_2_)(C_12_H_8_N_2_)(H_2_O)_2_]·2H_2_O, the Mn^II^ atom is coordinated by two N atoms from a 1,10-phenanthroline ligand, two water O atoms, a monodentate acetate anion and an *O*-monodentate 2-(1*H*-benzotriazol-1-yl)acetate ligand, resulting in a distorted *cis*-MnN_2_O_4_ octa­hedral coordination geometry. The water O atoms are in a *trans* arrangement and one of them forms an intra­molecular O—H⋯O hydrogen bond to the uncoordinated O atom of the acetate ion. In the crystal, the complex mol­ecules and water mol­ecules are connected by O—H⋯O and O—H⋯N hydrogen bonds to generate a three-dimensional network.

## Related literature

For related structures, see: Zheng *et al.* (2010 ▶); Zeng & Wang (2012 ▶).
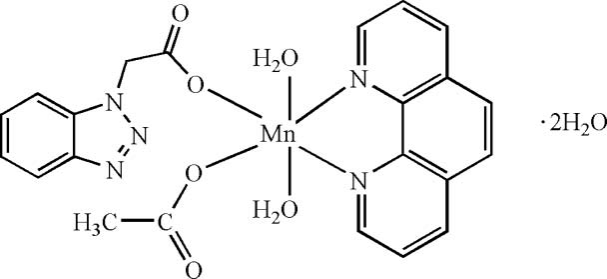


## Experimental

### 

#### Crystal data

[Mn(C_8_H_6_N_3_O_2_)(C_2_H_3_O_2_)(C_12_H_8_N_2_)(H_2_O)_2_]·2H_2_O
*M* *_r_* = 542.41
Orthorhombic, 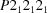

*a* = 6.877 (1) Å
*b* = 17.383 (3) Å
*c* = 20.033 (3) Å
*V* = 2394.9 (6) Å^3^
*Z* = 4
Mo *K*α radiation
μ = 0.61 mm^−1^
*T* = 296 K
0.22 × 0.18 × 0.16 mm

#### Data collection

Bruker APEXII CCD area-detector diffractometer
Absorption correction: multi-scan (*SADABS*; Bruker, 2005 ▶) *T*_min_ = 0.878, *T*_max_ = 0.909
16178 measured reflections
4247 independent reflections
3921 reflections with *I* > 2σ(*I*)
*R*_int_ = 0.029

#### Refinement

*R*[*F* ^2^ > 2σ(*F* ^2^)] = 0.027
*wR*(*F* ^2^) = 0.066
*S* = 1.00
4247 reflections
325 parameters
1 restraint
H-atom parameters constrained
Δρ_max_ = 0.20 e Å^−3^
Δρ_min_ = −0.16 e Å^−3^
Absolute structure: Flack (1983 ▶), 1798 Friedel pairs
Flack parameter: −0.028 (15)

### 

Data collection: *APEX2* (Bruker, 2005 ▶); cell refinement: *SAINT* (Bruker, 2005 ▶); data reduction: *SAINT*; program(s) used to solve structure: *SHELXS97* (Sheldrick, 2008 ▶); program(s) used to refine structure: *SHELXL97* (Sheldrick, 2008 ▶); molecular graphics: *SHELXTL* (Sheldrick, 2008 ▶); software used to prepare material for publication: *SHELXTL*.

## Supplementary Material

Crystal structure: contains datablock(s) global, I. DOI: 10.1107/S1600536812007404/hb6645sup1.cif

Structure factors: contains datablock(s) I. DOI: 10.1107/S1600536812007404/hb6645Isup2.hkl

Additional supplementary materials:  crystallographic information; 3D view; checkCIF report

## Figures and Tables

**Table:** 

Mn1—O1	2.1009 (14)
Mn1—O3	2.1522 (14)
Mn1—O5	2.2221 (16)
Mn1—O6	2.2807 (16)
Mn1—N4	2.2532 (16)
Mn1—N5	2.2935 (16)

**Table:** 

N4—Mn1—N5	73.09 (6)

**Table 2:** Hydrogen-bond geometry (Å, °)

*D*—H⋯*A*	*D*—H	H⋯*A*	*D*⋯*A*	*D*—H⋯*A*
O5—H23⋯N3^i^	0.85	1.99	2.838 (2)	173
O5—H24⋯O6^ii^	0.85	2.14	2.987 (2)	172
O6—H25⋯O8^iii^	0.85	1.88	2.732 (2)	175
O6—H26⋯O4	0.85	1.80	2.621 (2)	161
O7—H27⋯O4^ii^	0.85	1.97	2.807 (3)	166
O7—H28⋯O3	0.85	2.06	2.911 (2)	174
O8—H29⋯O7	0.85	2.04	2.890 (3)	176
O8—H30⋯O2^iv^	0.85	1.93	2.773 (2)	171

Symmetry codes: (i) 
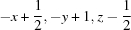
; (ii) 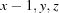; (iii) 
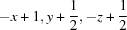
; (iv) 
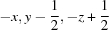
.

**Fig. 1.**
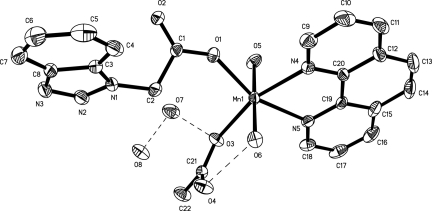
Molecular structure of the title compound with 40% probability displacement ellopsoids. Hydrogen bonds are shown as dashed lines).

## supplementary crystallographic information

### Comment

Construction of supramolecular architectures with intersting physical properties has grown rapidly owing to their potential use as new functional materials. Many intriguing supramolecular assemblies have been prepared by metal coordination or hydrogen bonding interactions. As a flexible ligand, 2-(1*H*-benzo[*d*][1,2,3]triazol-1-yl)acetic acid contains a carboxylate group and a triazole to construct MOFs (Zheng *et al.*, 2010). We have also been interested in this systems (Zeng *et al.*, 2012). As continuation of previous work, herein we report the synthesis and crystal structure of the title new complex (I).

As shown in Figure 1, The Mn(II) atom is six-coordinated by two N atoms from one 1,10-phenanthroline ligand, two O atoms from water molecules, one O atom from an acetate anion and one O atom from 2-(1*H*-benzo[*d*][1,2,3]triazol-1-yl)acetate anion in a distorted octahedral geometry (Table 1). The equatorial plane is defined by N4, N5, O1 and O3 with a mean deviation of 0.2357 (1) Å from the least-squares plane. The axial positions are occupied by O5 and O6 with an O5—Mn1—O6 angle of 179.46 (5) °. The Mn—O and Mn—N bond distance fall in range of 2.1009 (14) to 2.2935 (16) Å. The deprotonated 2-(1*H*-benzo[*d*][1,2,3]triazol-1-yl)acetic acid ligand adopt a monodentate coordination mode, which is different another manganese complex of this ligand (Zheng *et al.*, 2010). An extensive three-dimensional hydrogen-bonding network formed by classical O—H···O and O—H···N interactions between the title complex molecules and the uncoodinated water molecules consolidate the crystal packing(Table 2).

### Experimental

A mixture of Mn(Ac)_2_ (0.5 mmol), 2-(1*H*-benzo[*d*][1,2,3]triazol-1-yl)acetic acid (0.5 mmol) and 1,10-phenanthroline(0.5 mmol) was dissolved in water (30 ml) and methanol (10 ml). and the pH of the solution was adjusted to 6–7 with 0.2 *M* aqueous NaOH and the solution was stirred for 3 h at room temperature. The solution was flitered and the flitrate was allowed to stand at room temperature. After slow evaporation over 2 weeks, light yellow blocks were obtained.

### Refinement

All H atoms were placed in idealized positions (O—H = 0.85 Å and C—H = 0.93–0.97 Å) and refined as riding atoms with *U*_iso_(H) = 1.2*U*_eq_(C) and *U*_iso_(H) = 1.5*U*_eq_(O).

### Crystal data

**Table:**

[Mn(C_8_H_6_N_3_O_2_)(C_2_H_3_O_2_)(C_12_H_8_N_2_)(H_2_O)_2_]·2H_2_O	*F*(000) = 1124
*M**_r_* = 542.41	*D*_x_ = 1.504 Mg m^−^^3^
Orthorhombic, *P*2_1_2_1_2_1_	Mo *K*α radiation, λ = 0.71073 Å
Hall symbol: P 2ac 2ab	Cell parameters from 6090 reflections
*a* = 6.877 (1) Å	θ = 2.3–25.1°
*b* = 17.383 (3) Å	µ = 0.61 mm^−^^1^
*c* = 20.033 (3) Å	*T* = 296 K
*V* = 2394.9 (6) Å^3^	Block, light yellow
*Z* = 4	0.22 × 0.18 × 0.16 mm

### Data collection

**Table:**

Bruker APEXII CCD area-detector diffractometer	4247 independent reflections
Radiation source: fine-focus sealed tube	3921 reflections with *I* > 2σ(*I*)
Graphite monochromator	*R*_int_ = 0.029
φ and ω scans	θ_max_ = 25.0°, θ_min_ = 2.0°
Absorption correction: multi-scan (*SADABS*; Bruker, 2005)	*h* = −8→8
*T*_min_ = 0.878, *T*_max_ = 0.909	*k* = −20→17
16178 measured reflections	*l* = −23→23

### Refinement

**Table:**

Refinement on *F*^2^	Secondary atom site location: difference Fourier map
Least-squares matrix: full	Hydrogen site location: inferred from neighbouring sites
*R*[*F*^2^ > 2σ(*F*^2^)] = 0.027	H-atom parameters constrained
*wR*(*F*^2^) = 0.066	*w* = 1/[σ^2^(*F*_o_^2^) + (0.0345*P*)^2^ + 0.244*P*] where *P* = (*F*_o_^2^ + 2*F*_c_^2^)/3
*S* = 1.00	(Δ/σ)_max_ = 0.001
4247 reflections	Δρ_max_ = 0.20 e Å^−^^3^
325 parameters	Δρ_min_ = −0.16 e Å^−^^3^
1 restraint	Absolute structure: Flack (1983), 1798 Friedel pairs
Primary atom site location: structure-invariant direct methods	Flack parameter: −0.028 (15)

### Special details

**Table:**

Geometry. All e.s.d.'s (except the e.s.d. in the dihedral angle between two l.s. planes) are estimated using the full covariance matrix. The cell e.s.d.'s are taken into account individually in the estimation of e.s.d.'s in distances, angles and torsion angles; correlations between e.s.d.'s in cell parameters are only used when they are defined by crystal symmetry. An approximate (isotropic) treatment of cell e.s.d.'s is used for estimating e.s.d.'s involving l.s. planes.
Refinement. Refinement of *F*^2^ against ALL reflections. The weighted *R*-factor *wR* and goodness of fit *S* are based on *F*^2^, conventional *R*-factors *R* are based on *F*, with *F* set to zero for negative *F*^2^. The threshold expression of *F*^2^ > σ(*F*^2^) is used only for calculating *R*-factors(gt) *etc*. and is not relevant to the choice of reflections for refinement. *R*-factors based on *F*^2^ are statistically about twice as large as those based on *F*, and *R*- factors based on ALL data will be even larger.

### Fractional atomic coordinates and isotropic or equivalent isotropic displacement parameters (Å^2^)

**Table:**

	*x*	*y*	*z*	*U*_iso_*/*U*_eq_	
Mn1	0.48232 (4)	0.569128 (16)	0.144955 (13)	0.03039 (9)	
O1	0.3490 (3)	0.62223 (10)	0.22730 (6)	0.0460 (4)	
O2	0.2052 (2)	0.64743 (9)	0.32397 (7)	0.0447 (4)	
O3	0.5219 (2)	0.45338 (8)	0.18058 (7)	0.0418 (4)	
O4	0.8210 (2)	0.45172 (9)	0.22318 (9)	0.0517 (4)	
O5	0.1767 (2)	0.54730 (10)	0.11431 (7)	0.0458 (4)	
H23	0.1486	0.5276	0.0767	0.069*	
H24	0.0736	0.5646	0.1323	0.069*	
O6	0.7967 (2)	0.59177 (9)	0.17539 (7)	0.0424 (4)	
H25	0.8153	0.6291	0.2021	0.064*	
H26	0.8158	0.5514	0.1984	0.064*	
O7	0.1737 (3)	0.37049 (11)	0.22114 (9)	0.0654 (5)	
H27	0.0775	0.4010	0.2181	0.098*	
H28	0.2695	0.3973	0.2078	0.098*	
O8	0.1294 (3)	0.20584 (10)	0.23428 (9)	0.0627 (5)	
H29	0.1366	0.2544	0.2300	0.094*	
H30	0.0210	0.1928	0.2173	0.094*	
N1	0.4872 (3)	0.57294 (9)	0.39664 (7)	0.0361 (4)	
N2	0.4403 (3)	0.50889 (11)	0.43050 (9)	0.0429 (5)	
N3	0.4474 (3)	0.52448 (11)	0.49445 (9)	0.0434 (5)	
N4	0.5099 (3)	0.68006 (9)	0.08755 (7)	0.0368 (4)	
N5	0.5326 (3)	0.53668 (9)	0.03534 (7)	0.0339 (4)	
C1	0.3324 (3)	0.61825 (12)	0.29005 (9)	0.0336 (5)	
C2	0.4949 (4)	0.57245 (13)	0.32387 (9)	0.0488 (6)	
H2A	0.4886	0.5196	0.3086	0.059*	
H2B	0.6192	0.5933	0.3098	0.059*	
C3	0.5260 (3)	0.63196 (12)	0.43862 (9)	0.0341 (5)	
C4	0.5872 (4)	0.70775 (14)	0.42952 (13)	0.0484 (6)	
H4	0.6056	0.7289	0.3874	0.058*	
C5	0.6184 (4)	0.74899 (16)	0.48687 (15)	0.0603 (7)	
H5A	0.6593	0.7998	0.4833	0.072*	
C6	0.5913 (4)	0.71776 (19)	0.55020 (14)	0.0636 (7)	
H6A	0.6137	0.7485	0.5874	0.076*	
C7	0.5332 (4)	0.64393 (16)	0.55946 (11)	0.0537 (7)	
H7	0.5162	0.6234	0.6019	0.064*	
C8	0.5003 (4)	0.60032 (12)	0.50197 (9)	0.0373 (5)	
C9	0.5099 (4)	0.74978 (12)	0.11341 (12)	0.0505 (6)	
H9	0.5076	0.7546	0.1596	0.061*	
C10	0.5130 (5)	0.81644 (13)	0.07508 (15)	0.0637 (7)	
H10	0.5148	0.8646	0.0953	0.076*	
C11	0.5133 (5)	0.80988 (14)	0.00765 (15)	0.0626 (7)	
H11	0.5120	0.8539	−0.0188	0.075*	
C12	0.5157 (4)	0.73761 (13)	−0.02219 (11)	0.0485 (6)	
C13	0.5152 (5)	0.7250 (2)	−0.09288 (13)	0.0670 (8)	
H13	0.5122	0.7671	−0.1215	0.080*	
C14	0.5191 (5)	0.65418 (19)	−0.11858 (11)	0.0636 (8)	
H14	0.5179	0.6482	−0.1647	0.076*	
C15	0.5248 (4)	0.58742 (15)	−0.07722 (10)	0.0483 (6)	
C16	0.5316 (4)	0.51232 (17)	−0.10186 (11)	0.0578 (7)	
H16	0.5319	0.5036	−0.1477	0.069*	
C17	0.5376 (4)	0.45219 (16)	−0.05881 (13)	0.0545 (7)	
H17	0.5406	0.4020	−0.0747	0.065*	
C18	0.5393 (3)	0.46666 (13)	0.00980 (11)	0.0437 (6)	
H18	0.5455	0.4250	0.0389	0.052*	
C19	0.5254 (3)	0.59700 (12)	−0.00732 (9)	0.0338 (5)	
C20	0.5160 (3)	0.67305 (11)	0.01976 (9)	0.0341 (5)	
C21	0.6669 (3)	0.41917 (13)	0.20661 (9)	0.0349 (5)	
C22	0.6497 (4)	0.33410 (13)	0.21677 (14)	0.0557 (7)	
H22A	0.6840	0.3080	0.1762	0.084*	
H22B	0.5183	0.3215	0.2287	0.084*	
H22C	0.7359	0.3183	0.2519	0.084*	

### Atomic displacement parameters (Å^2^)

**Table:**

	*U*^11^	*U*^22^	*U*^33^	*U*^12^	*U*^13^	*U*^23^
Mn1	0.03638 (17)	0.03095 (16)	0.02383 (13)	0.00024 (15)	0.00013 (13)	0.00160 (11)
O1	0.0583 (11)	0.0556 (10)	0.0240 (7)	0.0128 (9)	0.0010 (7)	−0.0032 (7)
O2	0.0480 (10)	0.0552 (10)	0.0310 (8)	0.0156 (9)	0.0037 (7)	0.0004 (7)
O3	0.0390 (9)	0.0354 (8)	0.0509 (8)	0.0020 (8)	−0.0088 (8)	0.0104 (6)
O4	0.0420 (10)	0.0405 (10)	0.0726 (11)	0.0014 (8)	−0.0154 (9)	0.0022 (8)
O5	0.0341 (9)	0.0671 (11)	0.0362 (8)	−0.0008 (8)	−0.0004 (7)	−0.0153 (7)
O6	0.0447 (9)	0.0406 (9)	0.0419 (8)	−0.0057 (7)	−0.0029 (7)	−0.0001 (7)
O7	0.0470 (11)	0.0570 (11)	0.0921 (14)	0.0034 (10)	0.0073 (10)	0.0243 (11)
O8	0.0586 (12)	0.0496 (11)	0.0799 (12)	−0.0010 (9)	−0.0242 (10)	0.0122 (9)
N1	0.0415 (10)	0.0395 (10)	0.0273 (7)	0.0077 (13)	−0.0013 (8)	0.0017 (7)
N2	0.0453 (12)	0.0393 (11)	0.0441 (10)	0.0049 (9)	−0.0028 (9)	0.0036 (9)
N3	0.0438 (12)	0.0478 (12)	0.0387 (10)	0.0015 (9)	−0.0006 (9)	0.0117 (8)
N4	0.0414 (11)	0.0318 (9)	0.0371 (8)	0.0015 (10)	0.0050 (9)	0.0001 (7)
N5	0.0321 (10)	0.0371 (9)	0.0324 (8)	0.0040 (8)	0.0019 (8)	−0.0032 (7)
C1	0.0427 (13)	0.0319 (11)	0.0261 (10)	0.0007 (10)	0.0013 (9)	−0.0037 (9)
C2	0.0563 (15)	0.0632 (15)	0.0269 (9)	0.0230 (18)	0.0022 (11)	−0.0051 (9)
C3	0.0321 (12)	0.0371 (11)	0.0330 (9)	0.0049 (10)	0.0012 (9)	0.0013 (8)
C4	0.0407 (14)	0.0443 (15)	0.0603 (15)	0.0040 (11)	0.0029 (12)	0.0121 (12)
C5	0.0494 (17)	0.0433 (15)	0.0882 (16)	−0.0034 (13)	−0.0059 (15)	−0.0134 (14)
C6	0.0537 (17)	0.075 (2)	0.0617 (13)	0.0075 (15)	−0.0164 (13)	−0.0298 (13)
C7	0.0493 (16)	0.0760 (18)	0.0358 (11)	0.0113 (15)	−0.0073 (12)	−0.0072 (11)
C8	0.0317 (12)	0.0489 (12)	0.0315 (9)	0.0066 (12)	−0.0012 (10)	0.0035 (9)
C9	0.0559 (16)	0.0359 (13)	0.0599 (13)	−0.0002 (14)	0.0089 (14)	−0.0057 (10)
C10	0.0580 (18)	0.0285 (12)	0.105 (2)	−0.0003 (14)	0.0102 (19)	0.0030 (13)
C11	0.0507 (17)	0.0422 (14)	0.095 (2)	0.0008 (15)	0.0110 (17)	0.0320 (14)
C12	0.0333 (13)	0.0524 (14)	0.0600 (13)	0.0024 (13)	0.0046 (12)	0.0270 (11)
C13	0.0529 (17)	0.095 (2)	0.0532 (14)	−0.0038 (19)	0.0006 (15)	0.0428 (15)
C14	0.0526 (17)	0.108 (2)	0.0303 (10)	−0.007 (2)	0.0014 (12)	0.0226 (13)
C15	0.0337 (12)	0.0810 (18)	0.0302 (10)	−0.0027 (14)	0.0055 (10)	−0.0009 (10)
C16	0.0446 (16)	0.094 (2)	0.0348 (11)	−0.0011 (16)	0.0051 (12)	−0.0204 (13)
C17	0.0375 (13)	0.0660 (16)	0.0600 (14)	0.0016 (13)	0.0025 (12)	−0.0336 (13)
C18	0.0378 (14)	0.0416 (13)	0.0518 (12)	0.0042 (11)	−0.0007 (11)	−0.0119 (10)
C19	0.0247 (11)	0.0483 (12)	0.0283 (9)	−0.0001 (10)	0.0041 (9)	0.0031 (8)
C20	0.0269 (11)	0.0401 (11)	0.0354 (9)	0.0010 (11)	0.0015 (9)	0.0101 (8)
C21	0.0406 (13)	0.0348 (12)	0.0294 (10)	0.0048 (11)	0.0032 (9)	0.0034 (9)
C22	0.0520 (17)	0.0395 (14)	0.0756 (17)	0.0032 (12)	−0.0033 (14)	0.0150 (13)

### Geometric parameters (Å, º)

**Table:**

Mn1—O1	2.1009 (14)	C4—C5	1.371 (4)
Mn1—O3	2.1522 (14)	C4—H4	0.9300
Mn1—O5	2.2221 (16)	C5—C6	1.392 (4)
Mn1—O6	2.2807 (16)	C5—H5A	0.9300
Mn1—N4	2.2532 (16)	C6—C7	1.357 (4)
Mn1—N5	2.2935 (16)	C6—H6A	0.9300
O1—C1	1.264 (2)	C7—C8	1.397 (3)
O2—C1	1.218 (2)	C7—H7	0.9300
O3—C21	1.272 (3)	C9—C10	1.390 (3)
O4—C21	1.247 (3)	C9—H9	0.9300
O5—H23	0.8499	C10—C11	1.356 (4)
O5—H24	0.8499	C10—H10	0.9300
O6—H25	0.8500	C11—C12	1.391 (4)
O6—H26	0.8500	C11—H11	0.9300
O7—H27	0.8500	C12—C20	1.402 (3)
O7—H28	0.8501	C12—C13	1.433 (4)
O8—H29	0.8499	C13—C14	1.334 (4)
O8—H30	0.8500	C13—H13	0.9300
N1—N2	1.343 (2)	C14—C15	1.427 (4)
N1—C3	1.353 (3)	C14—H14	0.9300
N1—C2	1.459 (2)	C15—C16	1.396 (4)
N2—N3	1.310 (3)	C15—C19	1.410 (3)
N3—C8	1.376 (3)	C16—C17	1.356 (4)
N4—C9	1.318 (3)	C16—H16	0.9300
N4—C20	1.364 (2)	C17—C18	1.397 (3)
N5—C18	1.321 (3)	C17—H17	0.9300
N5—C19	1.354 (2)	C18—H18	0.9300
C1—C2	1.530 (3)	C19—C20	1.430 (3)
C2—H2A	0.9700	C21—C22	1.497 (3)
C2—H2B	0.9700	C22—H22A	0.9600
C3—C8	1.394 (3)	C22—H22B	0.9600
C3—C4	1.395 (3)	C22—H22C	0.9600
			
O1—Mn1—O3	101.87 (6)	C7—C6—C5	122.2 (2)
O1—Mn1—O5	83.06 (6)	C7—C6—H6A	118.9
O3—Mn1—O5	92.96 (6)	C5—C6—H6A	118.9
O1—Mn1—N4	93.52 (6)	C6—C7—C8	116.6 (2)
O3—Mn1—N4	163.47 (6)	C6—C7—H7	121.7
O5—Mn1—N4	94.87 (7)	C8—C7—H7	121.7
O1—Mn1—O6	97.35 (6)	N3—C8—C3	108.16 (17)
O3—Mn1—O6	87.29 (6)	N3—C8—C7	130.8 (2)
O5—Mn1—O6	179.46 (5)	C3—C8—C7	121.0 (2)
N4—Mn1—O6	84.77 (7)	N4—C9—C10	123.3 (2)
O1—Mn1—N5	157.76 (6)	N4—C9—H9	118.3
O3—Mn1—N5	93.93 (6)	C10—C9—H9	118.3
O5—Mn1—N5	80.56 (6)	C11—C10—C9	118.7 (2)
N4—Mn1—N5	73.09 (6)	C11—C10—H10	120.6
O6—Mn1—N5	98.95 (6)	C9—C10—H10	120.6
C1—O1—Mn1	142.76 (15)	C10—C11—C12	120.3 (2)
C21—O3—Mn1	132.21 (14)	C10—C11—H11	119.9
Mn1—O5—H23	121.9	C12—C11—H11	119.9
Mn1—O5—H24	127.7	C11—C12—C20	117.7 (2)
H23—O5—H24	109.1	C11—C12—C13	124.2 (2)
Mn1—O6—H25	116.2	C20—C12—C13	118.0 (2)
Mn1—O6—H26	98.5	C14—C13—C12	121.5 (2)
H25—O6—H26	105.5	C14—C13—H13	119.3
H27—O7—H28	103.8	C12—C13—H13	119.3
H29—O8—H30	106.1	C13—C14—C15	121.8 (2)
N2—N1—C3	111.23 (15)	C13—C14—H14	119.1
N2—N1—C2	120.56 (18)	C15—C14—H14	119.1
C3—N1—C2	128.20 (18)	C16—C15—C19	117.5 (2)
N3—N2—N1	108.26 (17)	C16—C15—C14	123.8 (2)
N2—N3—C8	108.36 (17)	C19—C15—C14	118.7 (2)
C9—N4—C20	118.25 (17)	C17—C16—C15	119.8 (2)
C9—N4—Mn1	125.90 (14)	C17—C16—H16	120.1
C20—N4—Mn1	115.74 (13)	C15—C16—H16	120.1
C18—N5—C19	118.07 (17)	C16—C17—C18	119.2 (2)
C18—N5—Mn1	127.06 (14)	C16—C17—H17	120.4
C19—N5—Mn1	114.11 (12)	C18—C17—H17	120.4
O2—C1—O1	126.6 (2)	N5—C18—C17	123.1 (2)
O2—C1—C2	119.59 (17)	N5—C18—H18	118.4
O1—C1—C2	113.76 (18)	C17—C18—H18	118.4
N1—C2—C1	114.38 (19)	N5—C19—C15	122.4 (2)
N1—C2—H2A	108.7	N5—C19—C20	118.57 (16)
C1—C2—H2A	108.7	C15—C19—C20	119.05 (19)
N1—C2—H2B	108.7	N4—C20—C12	121.69 (19)
C1—C2—H2B	108.7	N4—C20—C19	117.48 (16)
H2A—C2—H2B	107.6	C12—C20—C19	120.83 (18)
N1—C3—C8	103.98 (17)	O4—C21—O3	124.3 (2)
N1—C3—C4	133.98 (19)	O4—C21—C22	118.6 (2)
C8—C3—C4	122.0 (2)	O3—C21—C22	117.1 (2)
C5—C4—C3	115.6 (2)	C21—C22—H22A	109.5
C5—C4—H4	122.2	C21—C22—H22B	109.5
C3—C4—H4	122.2	H22A—C22—H22B	109.5
C4—C5—C6	122.6 (3)	C21—C22—H22C	109.5
C4—C5—H5A	118.7	H22A—C22—H22C	109.5
C6—C5—H5A	118.7	H22B—C22—H22C	109.5
			
O3—Mn1—O1—C1	25.8 (3)	N2—N3—C8—C3	0.1 (3)
O5—Mn1—O1—C1	117.4 (3)	N2—N3—C8—C7	−177.9 (3)
N4—Mn1—O1—C1	−148.1 (3)	N1—C3—C8—N3	−0.1 (3)
O6—Mn1—O1—C1	−63.0 (3)	C4—C3—C8—N3	−177.6 (2)
N5—Mn1—O1—C1	160.1 (2)	N1—C3—C8—C7	178.2 (2)
O1—Mn1—O3—C21	−96.90 (18)	C4—C3—C8—C7	0.7 (4)
O5—Mn1—O3—C21	179.57 (17)	C6—C7—C8—N3	177.7 (3)
N4—Mn1—O3—C21	61.4 (3)	C6—C7—C8—C3	−0.1 (4)
O6—Mn1—O3—C21	0.04 (17)	C20—N4—C9—C10	0.9 (4)
N5—Mn1—O3—C21	98.84 (18)	Mn1—N4—C9—C10	−175.1 (2)
C3—N1—N2—N3	0.0 (3)	N4—C9—C10—C11	1.0 (5)
C2—N1—N2—N3	178.9 (2)	C9—C10—C11—C12	−1.7 (5)
N1—N2—N3—C8	−0.1 (3)	C10—C11—C12—C20	0.5 (5)
O1—Mn1—N4—C9	22.2 (2)	C10—C11—C12—C13	179.7 (3)
O3—Mn1—N4—C9	−136.5 (2)	C11—C12—C13—C14	179.5 (3)
O5—Mn1—N4—C9	105.5 (2)	C20—C12—C13—C14	−1.3 (5)
O6—Mn1—N4—C9	−74.9 (2)	C12—C13—C14—C15	−0.3 (5)
N5—Mn1—N4—C9	−175.9 (3)	C13—C14—C15—C16	−179.1 (3)
O1—Mn1—N4—C20	−153.93 (19)	C13—C14—C15—C19	0.3 (5)
O3—Mn1—N4—C20	47.4 (3)	C19—C15—C16—C17	0.3 (4)
O5—Mn1—N4—C20	−70.61 (18)	C14—C15—C16—C17	179.7 (3)
O6—Mn1—N4—C20	108.99 (19)	C15—C16—C17—C18	−0.8 (4)
N5—Mn1—N4—C20	7.98 (17)	C19—N5—C18—C17	−0.6 (3)
O1—Mn1—N5—C18	−123.4 (2)	Mn1—N5—C18—C17	168.77 (17)
O3—Mn1—N5—C18	12.03 (19)	C16—C17—C18—N5	1.0 (4)
O5—Mn1—N5—C18	−80.32 (19)	C18—N5—C19—C15	0.1 (3)
N4—Mn1—N5—C18	−178.4 (2)	Mn1—N5—C19—C15	−170.66 (17)
O6—Mn1—N5—C18	99.90 (19)	C18—N5—C19—C20	179.4 (2)
O1—Mn1—N5—C19	46.3 (3)	Mn1—N5—C19—C20	8.6 (3)
O3—Mn1—N5—C19	−178.25 (15)	C16—C15—C19—N5	0.1 (4)
O5—Mn1—N5—C19	89.40 (15)	C14—C15—C19—N5	−179.4 (2)
N4—Mn1—N5—C19	−8.68 (15)	C16—C15—C19—C20	−179.2 (2)
O6—Mn1—N5—C19	−90.38 (15)	C14—C15—C19—C20	1.3 (4)
Mn1—O1—C1—O2	−161.02 (19)	C9—N4—C20—C12	−2.1 (4)
Mn1—O1—C1—C2	20.0 (4)	Mn1—N4—C20—C12	174.27 (19)
N2—N1—C2—C1	110.1 (2)	C9—N4—C20—C19	177.1 (2)
C3—N1—C2—C1	−71.2 (3)	Mn1—N4—C20—C19	−6.5 (3)
O2—C1—C2—N1	−4.1 (3)	C11—C12—C20—N4	1.4 (4)
O1—C1—C2—N1	174.9 (2)	C13—C12—C20—N4	−177.8 (3)
N2—N1—C3—C8	0.1 (3)	C11—C12—C20—C19	−177.7 (2)
C2—N1—C3—C8	−178.7 (2)	C13—C12—C20—C19	3.0 (4)
N2—N1—C3—C4	177.1 (2)	N5—C19—C20—N4	−1.6 (3)
C2—N1—C3—C4	−1.7 (5)	C15—C19—C20—N4	177.7 (2)
N1—C3—C4—C5	−177.2 (3)	N5—C19—C20—C12	177.6 (2)
C8—C3—C4—C5	−0.6 (4)	C15—C19—C20—C12	−3.1 (4)
C3—C4—C5—C6	0.0 (4)	Mn1—O3—C21—O4	7.5 (3)
C4—C5—C6—C7	0.5 (4)	Mn1—O3—C21—C22	−171.64 (16)
C5—C6—C7—C8	−0.4 (4)		

### Hydrogen-bond geometry (Å, º)

**Table:**

*D*—H···*A*	*D*—H	H···*A*	*D*···*A*	*D*—H···*A*
O5—H23···N3^i^	0.85	1.99	2.838 (2)	173
O5—H24···O6^ii^	0.85	2.14	2.987 (2)	172
O6—H25···O8^iii^	0.85	1.88	2.732 (2)	175
O6—H26···O4	0.85	1.80	2.621 (2)	161
O7—H27···O4^ii^	0.85	1.97	2.807 (3)	166
O7—H28···O3	0.85	2.06	2.911 (2)	174
O8—H29···O7	0.85	2.04	2.890 (3)	176
O8—H30···O2^iv^	0.85	1.93	2.773 (2)	171

Symmetry codes: (i) −*x*+1/2, −*y*+1, *z*−1/2; (ii) *x*−1, *y*, *z*; (iii) −*x*+1, *y*+1/2, −*z*+1/2; (iv) −*x*, *y*−1/2, −*z*+1/2.
